# 2234 Developing the future translational science workforce at the University of Iowa

**DOI:** 10.1017/cts.2018.203

**Published:** 2018-11-21

**Authors:** James Torner, Beth R. Knudson, Kimberly Dukes

**Affiliations:** Institute for Clinical and Translational Science, University of Iowa

## Abstract

OBJECTIVES/SPECIFIC AIMS: To evaluate the extent to which the curriculum delivered via an innovative program, the Early Scholars Certificate in Clinical and Translational Science (CCTS) at the University of Iowa (UI), develops a translational science workforce pipeline by increasing awareness of and interest in translational science as a career goal for highly prepared undergraduates. METHODS/STUDY POPULATION: The CCTS’s objective is to increase the awareness of the philosophy and tools of translational science and to incorporate critical evaluation and self-appraisal of the translational aspects of a scholar’s own research. CCTS is a 16-semester-hour (sh) academic certificate program introducing translational science concepts and careers to undergraduate students. The CCTS is a selective program with requirements including a minimum GPA, minimum sh completed, completion of course prerequisites, and already engaged and supported by mentored research. The curriculum includes electives in the area of their research interests (6 sh); graduate level Epidemiology (3 sh); Biostatistics (3 sh); and 2 core Translational Research courses (4 sh total). The first core course, an Introduction to Translational Research, is a survey course providing students the opportunity to learn how translational research is conceived and developed. It is designed to instruct the student how to interpret their research in a translational T1 to T4 paradigm. The program’s capstone course, Practicum in Translational Research, provides undergraduate students the opportunity to address how their research experience translates into clinical practice. Student’s spend the majority of this course’s contact hours in a shadowing experience with a clinician in the area of their research. Students reflect on this shadowing experience and its relevance to their academic and professional goals. The students also spend time developing skills in peer review—not only learning to provide constructive feedback to other research professionals, but also how to receive and integrate the feedback. The course includes a mock research fair where both UI faculty and classmates provide feedback that is later integrated into their capstone projects—a poster presentation at the UI Carver College of Medicine Research Fair as well as a final translational paper. As part of the ongoing evaluation of the program and graduates, we examined the participant data, the course satisfaction with content, the change in understanding of translational science, and the intention to incorporate translational science into research and career goals. We also conducted course evaluation surveys and qualitative analysis of a focus group and interviews. RESULTS/ANTICIPATED RESULTS: Since 2015, the CCTS program has introduced translational science curriculum to 20 undergraduate participants (men/woman 40%/60%; 5% Hispanic or Latino; 15% Center for Diversity and Enrichment Eligible). Areas of academic interest include: biology, genetics, engineering, bioinformatics, biochemistry, neuroscience, psychology, and microbiology. Graduates of the Certificate and degree program to date (n=8) have gone onto: Fullbright awards (1), medical school/Masters in public health (1), combined MD/PhD programs (2), biomedical PhD program (1), or currently work in translational science positions in industry (2). In questionnaire and focus group results, we found that in general, students reported increased understanding of the translational spectrum and felt the certificate program helped them clarify their educational or career goals. Data from both the focus group and the questionnaire demonstrate that students are strongly positive about the program in general, including its quality, faculty and guest speakers, structure, goals, opportunities, personality, and personnel. All students highly valued many elements of the program and each course, and particularly the opportunity for clinical shadowing. Among the questionnaire findings for 2016–17, all students (100%) rated program quality “excellent,” and 7 of 8 (87.5%) “strongly agreed” that they better understood translational science, that they saw themselves continuing in translational science research after graduation, and they were better able to communicate how their lab research fits within the translational spectrum. In each case 1 of 8 “agreed.” Participants also generally felt that their career goals had been affirmed or realigned, and that they were better able to communicate the meaning of translational science to multiple audiences. Responses on changes to career aspirations and plans were mixed, and are ambiguous. Questionnaire Item 4, “My UI curricular and/or co-curricular plans changed as a result of the CCTS program,” which had mixed responses, asked specifically about the CCTS program as a reason for change, but it is not clear if, whether, or how the program specifically wants to change curricular plans. In the focus group, students reported using their individual shadowing and lab experience in determining preferences and intentions about future career choices (e.g., whether or not to apply to medical school and/or pursue basic science research). Participants perceived the shadowing experience, complementing or contrasting their lab research, as particularly relevant in deciding about their future careers. Other themes that emerged from the focus group and/or open section of the questionnaire demonstrate the impact of various course elements on participants’ understanding of translational science and potential careers, including: quality of instruction, program and course content (including guest speakers, the shadowing experience, and the poster development process); the exposure to a range of possibilities along the translational spectrum and the expansion of ideas about what research could look like; the value of connections (to faculty, researchers and clinicians, and other CCTS students and alumni); the attributes of the cohort; and the “personality” of the program and personnel. DISCUSSION/SIGNIFICANCE OF IMPACT: Developing a pipeline for translational science workforce development has been problematic because a lack of the understanding of the need of translational research and a structuring a time efficient program for early career clinical and basic scholars. Undergraduates making critical decisions about educational paths and career goals and plans may not be aware of opportunities in translational science or the type of choices they need to make to prepare for such opportunities. Our data demonstrates that CCTS was an effective way of introducing translational science concepts and career paths to undergraduate students and potentially a powerful way to encourage them to consider these career paths. Participants in our program improved their knowledge of the field and expressed interest and intention to incorporate translational science training into their career plans. However, improvements can be made in the CCTS program. Additionally, CTSAs should consider ways to incorporate findings like these into a wider sphere of training to help develop and strengthen a translational science workforce for the future. The exposure to a variety of translational science career possibilities and specialties was important to students. Based on both focus group discussion and questionnaire data, a few students did expand slightly their sense of career possibilities, but the larger benefit may be their concrete experiences that validate or solidify their interests, making them more skilled at talking about and supporting their career goals on applications and in interviews. Shadowing did not always encourage students to go into clinical medicine, but often solidified interests or leanings students already had, giving them a more grounded basis for refining their decisions. For some students, shadowing a clinician confirmed ideas of being a physician; for others, it steered them away from it. Some now found ethical challenges, bureaucracy, or emotional challenges daunting or newly necessary to consider before focusing on clinical careers. This may be just what students need at this point, and emphasizes for them the relation between different kinds of research and application within translational science. Our evaluation suggests that CCTS contributes to academic choices for career development and additionally can help attract highly skilled students into TS research, including students of color. Future work to evaluate CCTS impact on graduates’ career outcomes will inform the translational research direction and content. In terms of program design, it could be useful to build in multiple opportunities for students to understand the diversity of translational science careers and provide students more exposure to different possibilities in clinical and translational work.

